# Mouse Models of 22q11.2-Associated Autism Spectrum Disorder

**DOI:** 10.4172/2165-7890.S1-001

**Published:** 2012-09-05

**Authors:** Noboru Hiroi, Takeshi Hiramoto, Kathryn M. Harper, Go Suzuki, Shuken Boku

**Affiliations:** 1 Department of Psychiatry and Behavioral Sciences, Albert Einstein College of Medicine, Golding 104, 1300 Morris Park Avenue, Bronx, NY, 10461 USA; 2 Department of Neuroscience, Albert Einstein College of Medicine, Golding 104, 1300 Morris Park Avenue, Bronx, NY, 10461 USA; 3 Department of Genetics, Albert Einstein College of Medicine, Golding 104, 1300 Morris Park Avenue, Bronx, NY, 10461 USA; 4 Department of Psychiatry & Behavioral Sciences, Northwestern University, Ward Building Room 9-258, 303 E. Chicago Ave. Chicago, IL 60611, USA; 5 Department of Psychiatry, National Defense Medical College, 3-2 Namiki, Tokorozawa, Saitama 359-8513, Japan

**Keywords:** *Tbx1*, *Sept5*, 22q11.2, Syndromic ASD, Copy number variation

## Abstract

Copy number variation (CNV) of human chromosome 22q11.2 is associated with an elevated rate of autism spectrum disorder (ASD) and represents one of syndromic ASDs with rare genetic variants. However, the precise genetic basis of this association remains unclear due to its relatively large hemizygous and duplication region, including more than 30 genes. Previous studies using genetic mouse models suggested that although not all 22q11.2 genes contribute to ASD symptomatology, more than one 22q11.2 genes have distinct phenotypic targets for ASD symptoms. Our data show that deficiency of the two 22q11.2 genes*Tbx1* and *Sept5* causes distinct phenotypic sets of ASD symptoms.

## Introduction

Genes are currently the best available entry point for the studies aimed at understanding the brain mechanisms underlying autism spectrum disorders (ASD). Early twin studies of ASD indicated the proportion attributable to genetic factors at about 90% [1–5]. Although a more recent, large-scale study with recent ASD criteria has estimated a lower rate of ASD heritability [6], it is still safe to conclude that genetic variation confers a considerable risk for ASD.

In an attempt to identify individual contributory genes, various types of genetic variants are being examined. Genome-wide association with many single nucleotide polymorphisms (SNPs) suggest that commonly found variants confer a 1.2–3 fold increase in risk for ASD [7]. Additionally, rare and genetically identifiable cases of ASD or syndromic ASDs are being explored. They include mutations of single genes and copy number variations (CNVs). While it remains unclear if rare variants and common variants seen in ASD share alterations in similar or overlapping molecular cascades and networks, rare variants are often associated with substantially increased risk for ASD than common variants, and thus study of rare variants is the best currently available approach towards identification of ASD mechanisms.

### 22q11.2 CNV represents a Syndromic ASD

Our group has focused on human chromosome 22q11.2 as a reliable genetic risk factor for ASD. Deficits in social behavior, skills and cognition have long been noted in 22q11.2 hemizygous children [8–15]. Fourteen to 50% of individuals with 22q11.2 hemizygosity examined for ASD are reported to meet diagnostic criteria [12,16–21]. Patients with 22q11.2 duplication meet criteria for ASD when evaluated using the Autism Diagnostic Observation Scale (ADOS), Autism Behavior Checklist (ABC), and Childhood Autism Rating Scale (CARS) [22–25]. However, patients with 22q11.2 CNV are often referred for formal psychiatric evaluation only after they exhibit cognitive, social and behavioral problems (i.e., ascertainment bias). Moreover, the number of duplication cases so far identified is not large enough to permit computation of the true rate of ASD. Nevertheless, when screened from the general ASD population, 22q11.2 hemizygosity and duplications have been identified as rare variants in many studies [26–34].

### 22q11.2 CNV and other Neuropsychiatric Disorders

Individuals with 22q11.2 hemizygosity exhibit other neuropsychiatric disorders, including severe, mild and borderline mental retardation (50–90%) [12,13,35–39], attention-deficit/hyperactivity disorder (35–55%) [12,38,40–44], obsessive compulsive disorder (8–33%) [41–45], schizophrenia (~25%) [35,43,45–52], generalized anxiety disorders (10–28.6%) [12,42,43], schizoaffective disorder (2–8%) [41,44,45,48], and other behavioral problems as well as phobias and anxiety disorders [12,40–45].

Karayiorgou and colleagues [53] pointed out that ASD and most diagnoses noted above, except schizophrenia, might not be genuinely associated with 22q11.2 hemizygosity. It is true that high rates (~25%) [50] of schizophrenia are associated with 22q11.2 hemizygosity [35,43, 45,47,48,54]. Clearly, more evidence is needed to associate 22q11.2 hemizygosity with additional diagnoses. However, ASD diagnosis was made by experienced raters and psychiatrists based on validated and reliable scales, such as the Autism Diagnostic Interview—Revised (ADI-R) and Diagnostic and Statistical Manual of Mental Disorders-IV (DSM-IV), in studies that reported higher than expected rates of ASD [16–20]. It is premature to dismiss the notion that heightened rates of ASD also are associated with 22q11.2 CNV.

While some subtle differences have been noted in symptomatic elements between a small sample of children with 22q11.2 hemizygosity and idiopathic autistic children [19], it is unclear if such subtle differences in a small sample size invalidate the ASD diagnosis given the generally variable nature of symptomatic presentation in idiopathic ASD. Similarly, Eliez [55] reported that children with hemizygosity exhibit language impairment but catch up following surgical and therapeutic interventions of cleft palate and their verbal reasoning skills are stronger than those for nonverbal reasoning; idiopathic autism is associated with weaker verbal profiles compared to nonverbal profiles throughout development. Only 10 of 300 children exhibited impaired verbal abilities among Eliez’s sample with 22q11 hemizygosity. However, language impairments are variable in 22q11.2 hemizygous babies and children[55]; although a majority show lower performance IQ than verbal IQ, a sizable subpopulation shows the reverse pattern [56]. Moreover, as children with 22q11.2 grow, verbal IQ declines more rapidly than performance IQ and verbal IQ becomes lower than or comparable to performance IQ [54, 57]. Idiopathic ASD children also have varying degrees of language delays [58, 59] and importantly, many of those with language delays become fluent speakers by later school years [60].

It has also been suggested that underlying processes (e.g., social motivation and social skills) may be different between 22q11.2 associated and idiopathic ASDs. Although individuals with idiopathic ASD are impaired in both motivation for social interaction with others [61–63] and processing of social cues and understanding the mental state of others (known as theory of mind) [64,65], a high degree of heterogeneity is noted and in fact, some display genuine signs of social motivation but lack the skills [61]. Thus, these processes do not provide a clear-cut discriminating power to differentiate between idiopathic and 22q11.2-associated ASD.

Although most of 22q11.2-associated neuropsychiatric disorders are not found at a higher frequency among individuals with the 22q11.2 microdeletion than in cohorts with other developmental disorders associated with learning disabilities [53], it should be noted that in the idiopathic ASD population, ASDs are associated with high rates of comorbidity with severe cognitive impairments [66–69] and intellectual disabilities [70]. Similarly, individuals with 22q11.2-associated ASD have high rates of developmental delays and cognitive impairments [12,13,35–39,71,72]. Given this comorbidity, it is not certain if there is a specific brain development and functional mechanism that is so selectively affected that only ASD is manifested without comorbidity.

It is true that a significant enrichment for 22q11.2 deletions was not found in ASD samples in some studies [53]. Ogilvie and colleagues reported no case with 22q11.2 deletion among 103 ASD patients from multiplex families [73], but this sample size is not sufficient for detection of a rare CNV. In another study of simplex and multiplex ASD cases, 22q11.2 duplications, but not hemizygosity, were enriched [74]. However, many other studies reported enrichment for 22q11.2 duplications and hemizygosity in ASD samples [26–33], and a combined analysis of studies with stringent criteria demonstrated statistically significant enrichment of 22q11.2 CNV in 3,816 ASD samples [34]. Statistically significant enrichment of any rare CNV is generally difficult to achieve after correction for multiple comparisons, due to its very rare nature [34]. Detection of 22q11.2 hemizygosity in ASD samples in simplex and multiplex cases is additionally complicated by the relatively higher rates of *de novo* as opposed to inherited hemizygosity [75–78] and the opposite trend for duplications [79–82].

It was suggested that diagnoses of ASD might reflect misdiagnosis of social impairments actually associated with premorbidity in schizophrenia [53]. Eliez [55] reported that 56% of children with childhood-onset schizophrenia are first diagnosed with pervasive development disorder (PDD), while rates for diagnosis of autism during childhood and schizophrenia later in life are less than 5%. However, one retrospective analysis indicates that half of schizophrenic patients meet the genuine diagnostic criteria for ASD during childhood [83]. More work is needed to dismiss the possibility that 22q11.2 hemizygosity increases susceptibility to both schizophrenia and ASD.

### Mouse Models of 22q11.2 CNV

It has not been feasible to ascertain the impact of dose alterations of individual 22q11.2 genes within the 1.5–6Mb CNV region on various phenotypes in humans. Association of single nucleotide polymorphisms (SNPs) on the remaining copy of 22q11.2 in individuals with ASD determines how such alleles modify phenotypes of 22q11.2 hemizygosity, but does not identify genes whose hemizygosity causes phenotypes. Moreover, SNPs are not equivalent to deletions or duplications and do not consistently confer susceptibility to neuropsychiatric disorders [84].

**Figure 1:**
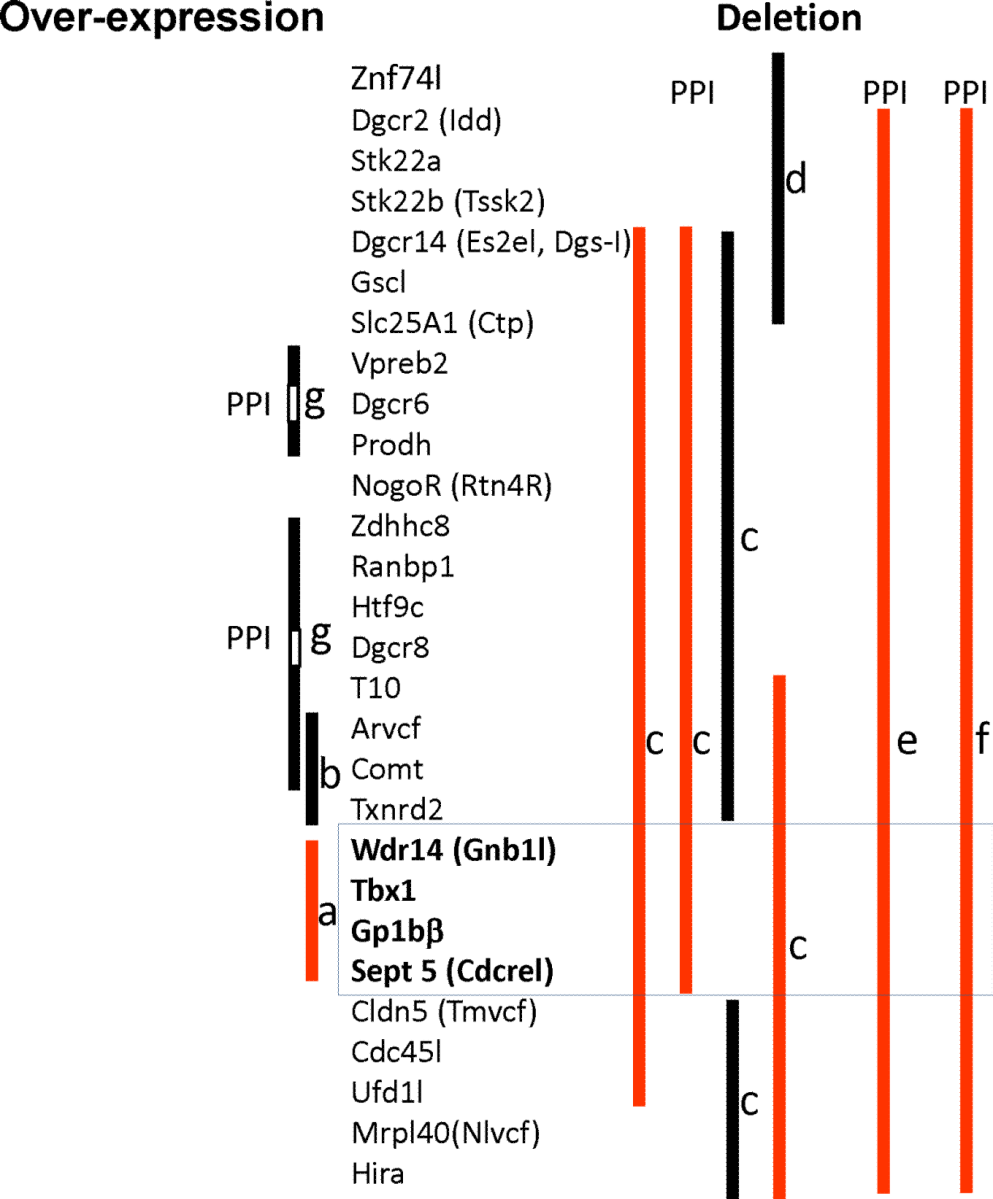
Genetic mouse models of 22q11.2 CNVs Over-expression (left) and deletion (right) cases are indicated. Vertical bars indicate the extent of chromosomal segments over-expressed or deleted. Phenotypes consistent (red) and inconsistent (black) with those associated with ASD are shown. a) hyperactivity, sensitization, social behaviors and clozapine-response are measured [85]; b) social interaction, working memory, prepulse inhibition (PPI), and anxiety and motor behavior were measured [93]; c,d,e,f and g) auditory PPI was measured. c[97], d[98], e[99], f[100], and g[94].

Modeling genetic abnormalities of 22q11.2 CNVs is relatively straightforward due to conserved sequence homology between the mouse and human. The usefulness of a rodent model resides in its ability to precisely manipulate a specific gene in isolation and predict its outcome; this is not possible in humans because human studies are, in essence, observation of correlation. We and others have used genetically engineered mouse models to identify small segments and single 22q11.2 genes responsible for ASD-related behavioral phenotypes (Figure 1).

It is inherently difficult to behaviorally model ASD symptoms in mice and any attempt to model symptoms in experimental animals is at best a proxy for the real behaviors/symptoms. While modeling overall symptomatology is difficult, ASD may be more reliably characterized when a link is sought between genetic risk factors and dimensions of a specific behavioral element of ASD. We have measured specific behavioral elements of ASD, including social interaction, social communication and repetitive behavior. Ultimate validation of the efficacy of mouse models will only be accepted when hypothetical mechanisms of ASD and therapeutic effectiveness in an animal model are consistent with observations in humans.

What has emerged from these mouse studies is the knowledge that not all 22q11.2 genes contribute to ASD-related behavioral phenotypes. In 2005, our group reported that mice over-expressing a ~200 kb segment of human 22q11.2, containing *Gnb1l, Tbx1,* Gp1B*β* and *Sept5*, exhibit hyperactivity, spontaneous sensitization, lack of normal social interaction (Figure 1a; see also Supporting Information, Movie 2 in [85]). Spontaneous sensitization of hyperactivity was completely blocked after three weeks of treatment with the antipsychotic drug clozapine [85]; clozapine and related atypical antipsychotic drugs attenuate some ASD symptomatic elements [86]. These phenotypes were present as early as 5 weeks old and persisted up to 2–4 months of age. However, the level of hyperactivity in this mouse model was so high that it might have rendered mice physically unable to engage in reciprocal social interaction. It was not technically possible to analyze more detailed affective and cognitive behaviors due to the extraordinarily high levels of hyperactivity.

We subsequently demonstrated that over-expression of an adjacent ~190 kb segment, containing *Arvcf, Comt* and *Txnrd2,* impaired working memory (consistent with deficits seen in 22q11.2 hemizygous patients [11,87–91] and idiopathic ASD patients [92]), but had no effect on reciprocal social interaction or prepulse inhibition (PPI) [93] (Figure 1b). However, working memory has not been examined in 22q11.2 duplication patients so far, and relevance of this mouse phenotype to duplication phenotypes remains unclear. The fact that this chromosomal segment dissociated working memory from PPI and social interaction suggests that these behavioral phenotypes are genetically dissociable.

Stark and colleagues provided complementary evidence that over-expression of chromosomal segments outside the 200 kb region does not induce PPI deficits [94]. Mice overexpressing a segment containing *Prodh* and *Vpreb2* exhibited a *higher* level of PPI than WT mice (Figure 1g). It is not clear whether this mouse phenotype is consistent with that in humans, because, to date, PPI has not been examined in duplication cases. Moreover, given that both 22q11.2 duplication and hemizygosity are associated with ASD, it might be expected that high and low doses of 22q11.2 cause phenotypes in the same, not opposite, direction. A second mouse line had over-expression of a segment that included *Zdhhc8, Ranbp1, Htf9c, T10, Arvcf* and *Comt* (Figure 1g); this mouse was indistinguishable in PPI from WT mice. This was consistent with our own data showing that the 190 kb transgenic mouse over-expressing COMT and two other genes showed normal PPI (Figure 1b) [93]. Similarly, Weinberger’s group demonstrated that Comt over-expression or deletion does not affect PPI [95]. Given that Comt elevation nevertheless impairs working memory in these mice [93,95], elevated levels of this 22q11.2 gene seem to selectively impair working memory without impacting PPI [93,95] or social interaction [93].

Taken together, these observations suggested that the 200 kb segment we identified (Figure 1a) [85] might contain a gene or genes that contribute to behavioral phenotypes related to ASD. The fact that over-expression of the 200 kb region alone was sufficient to induce behavioral phenotypes related to ASD is of considerable interest, as it implies that this genomic abnormality could act as a primary causative event rather than a susceptibility factor. Note that the phenotypic targets of individual 22q11.2 genes are not identical. Over-expression of the 200 kb region causes a number of behavioral phenotypes related to ASD, whereas that of the adjacent 190 kb region results in selectively impaired working memory.

Children with 22q11.2 hemizygosity exhibit defective auditory PPI [96]. The genetic origin of this behavioral phenotype was identified by a series of elegant mouse studies. Several groups examined the effects on PPI of 1.5 Mb or smaller, partly overlapping deletions of murine chromosome 16, a mouse ortholog of human 22q11.2 (Figure 1). Auditory PPI was defective only when large deletions encompassed the same 200 kb region; when large deletions occurred outside the 200 kb region, no PPI deficit was seen [97–100] (Figure 1c,d,e and f). These reports conclusively demonstrated that the same 200 kb region is also responsible for this behavioral phenotype in 22q11.2 hemizygosity.

Collectively, these mouse studies form a solid basis upon which to further study genetic mechanisms of 22q11.2-associated ASD. Our subsequent studies have focused on two genes encoded in the 200 kb region in mouse models.

#### Tbx1

A rare case of *TBX1* mutation (not 22q11.2 hemizygosity) was associated with Asperger syndrome in one individual [97]. *Tbx1* is one of four genes encoded in the 200 kb region and belongs to a phylogenetically conserved family of genes that share a common DNA-binding domain, the T-box. The human TBX1 protein and its mouse ortholog Tbx1 share a highly conserved amino acid sequence. *Tbx1* mRNA is present at low levels in the embryonic mouse brain and is expressed at increasingly higher levels in the postnatal and adult mouse brain [97].

Reverse transcription-polymerase chain reaction (RT-PCR) analysis showed *Tbx1* mRNA expression in the prefrontal cortex, nucleus accumbens, caudate-putamen, amygdala, hippocampus, ventral tegmental area, and substantia nigra of C57BL/6J mice at 2 months of age [101]. Immunofluorescent analysis similarly showed that low signal levels of *Tbx1* were present in many brain regions of 2 month-old C57BL/6J mice, but higher levels were found in the rostral migratory stream, the dentate gyrus, and the subventricular zone. These data are consistent with the reports that *Tbx1* mRNA and protein are present in the whole adult mouse brain samples [97,102], and further reveal the presence of *Tbx1* mRNA and protein in distinct brain regions. Interestingly, these brain regions are known to undergo postnatal and adult neurogenesis. In fact, higher *Tbx1* protein levels have been reported during proliferation than differentiation in neural progenitor cell cultures derived from the hippocampal dentate gyrus [101].

Note that *Tbx1* has been deposited as an alias of mouse lipopolysaccharide-induced TNF factor (*Litaf*) at one NCBI site (GenBank: AF171100.1; http://www.ncbi.nlm.nih.gov/nuccore/AF171100) despite the fact that these two genes have different sequences and different chromosomal locations (*Tbx1*, *Mus musculus* chromosome 16, 18581713-18586969; *Litaf, Mus musculus* chromosome 16, 10959273-10993121). This error has propagated other *Tbx1* and Litaf listings on the NCBI, MGI and many other similar sites and might be a reason why one published comprehensive analysis of 22q11 gene expression used “*Tbx1*” primers that have no sequence homology with *Tbx1* and reported that “*Tbx1*” mRNA signals, which are in reality litaf signals, were not detectable in any brain regions of adult mice.

Although, we noted sensitized hyperactivity in 200 kb transgenic mice (Figure 1a) [85], relevance of this behavior to ASD is also not clear. While clozapine attenuated hyperactivity is caused by over-expression of the 200 kb [85] segment and it is known that this drug attenuates some symptoms of ASD [86], it is unclear whether sensitized hyperactivity in mice models the core symptoms of ASD.

While PPI is a reliable parameter for sensorimotor gating [103], its relevance to ASD has not been definitively established. Defective PPI is not consistently seen in individuals with ASD [104–107]. Moreover, evidence suggests that PPI, as an endophenotype, is genetically dissociable from symptomatic elements of ASD and schizophrenia. For example, in *Sept5* KO mice, social interaction is reduced but PPI is potentiated [108–110].

**Figure 2:**
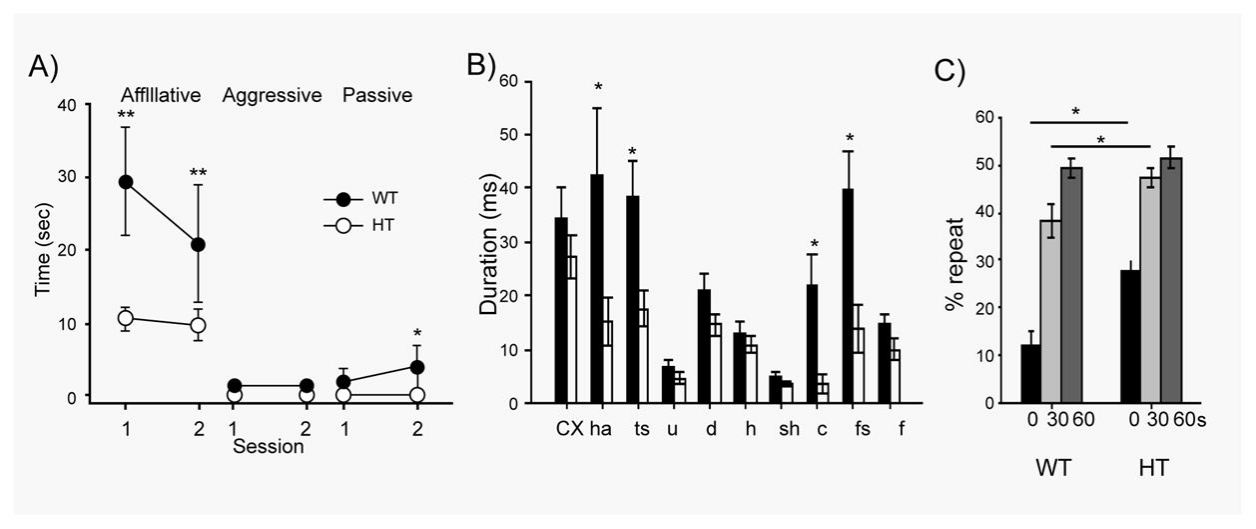
A) Active affiliative (affiliative), aggressive, and passive affiliative (pasasive) forms of social interaction in*Tbx1 WT* and HT mice Time spent(mean ± SEM) in the three forms of social interaction in two 5-min sessions with an age-matched stimulus C57BL/6J mouse is shown. Asterisks indicate statistically significant differences between WT and HT mice at levels of 0.05(*) and 0.01(**), as determined by Newman-Keuls comparisons. B) Ultrasonic vocalization of pups during a 5-min separation from mothers at postnatal days 7–8. The average duration (mean±SEM) of each vocal call type is shown. Distinct catagories of calls, as defined by Scattoni and colleagues [138], are indicated as: cx, complex; ham, harmonics; ts, two syllable; u, upward; d, downward; h, hump(a.k.a., chevron); sh, shorts; c, composite; fs, frequency steps; f, flat. An asterisk indicates a statistically significant difference between WT and HT mice at levels of 0.05(*) and 0.01 (**) as determined by Newman-Keuls comparisions. C) Spontaneous alternation in T-maze. The percentage of repeated visits to the same am (mean ± SEM) is shown. Mice were tested with 0-, 30-, and 60-s delays between trails. An asterisk indicates a statistically significant difference between WT and HT mice at 0.01 (**) at each delay (solid line), as determined by Newman-Keuls comparisions. This figure is reproduced from [101] with permission of the Oxford Press.

We, thus, examined social interaction in a naturalistic social interaction paradigm in which an age-matched, male C57BL/6J inbred mouse was paired with either a congenic *Tbx1* HT mouse or WT mouse; *Tbx1* homozygous mice are not viable. As a pair of mice is placed in a cage that is novel to both, there is no ‘resident’ mouse in this task; aggressive social interaction is minimized and affiliative social interaction is maximally evaluated [93,108]. Unlike a “sociability” task in which one of the mice is confined in a small wire cage, reciprocal interaction can be evaluated in this naturalistic social interaction task [111]. *Tbx1* HT mice exhibited significantly lower levels of active and passive affiliative social interaction (Figure 2A); no detectible aggressive social behavior was seen in this setup.

Babies and children with 22q11.2 hemizygosity exhibit delayed development of vocal volume, vocalization, and language [112] and social communication deficits [8–21]. When mouse pups are separated from mothers, they typically emit ultrasonic vocalization. This vocalization elicits their retrieval by mothers, and thus is considered a form of social communication in rodents [113,137]. We examined ultrasonic vocalization at postnatal days 7–8. HT mice exhibited vocalization for shorter duration in harmonic, two-syllable, composite, and frequency steps, compared to WT littermates (Figure 2B). Interestingly, these defective vocalization patterns in HT mice are fairly complex, but WT and HT vocalizations were indistinguishable in simple patterns (e.g., upward, downward, hump, and short).

Mice have a natural tendency to alternate arms visited in a T-maze, a behavior that requires working memory to recall a previously arm visited and alternate visits [114]. *Tbx1* HT mice showed higher levels of repeated visits to the same arm (Figure 2C). When HT mice showed working memory at 0 seconds delay, they had a higher degree of repetitive choices than WT mice; at a 60-sec delay, HT and WT mice were indistinguishable in repetitiveness and HT mice did not show increased repetitiveness beyond 50% (Figure 2C). These data suggest that the repetitive behavioral tendency is present in HT mice only when it depends on working memory, and is not indicative of simple motor repetitiveness. It is interesting to note that individuals with idiopathic ASD have difficulty in inhibiting context-inappropriate behavior based on working memory; this is thought to underlie actions and verbalizations that are inappropriate in terms of timing or appropriateness to the circumstances; they are not impaired in simple response inhibition that is not dependent on memory [115,116]. Taken together, *Tbx1* heterozygosity recapitulates symptomatic features of 22q11.2-associated ASD.

#### Sept5

*Sept5* is abundantly expressed in rodent and human brains [117,118], and is presynaptically located to regulate neurotransmitter release at synapses together with the SNARE complex [119–121]. This protein additionally contributes to the structural health of axons and dendrites [122,123]. Given that synaptic alterations in synaptic and neuronal connection are seen in many mouse models of ASD, and our results showed that a gene dose alteration of the 200 kb region, including *Sept5*, impaired social interaction [85], we evaluated two issues regarding the functional role of *Sept5* in ASD-related behavioral phenotypes.

Firstly, since not all individuals with 22q11.2 hemizygosity show ASD (i.e., incomplete penetrance) [12,16, 21], we hypothesized that genetic background affects phenotypic expression of *Sept5* deficiency. Second, while limbic region activation occurs when humans are exposed to social cues and this activation is altered in individuals with ASD, the genuine functional role of these alterations in ASD and the brain regions through which *Sept5* functionally mediates social behavior are not known. We hypothesized that *Sept5* levels in the two major limbic regions (hippocampus and amygdala) are a determinant of social interaction.

**Figure 3:**
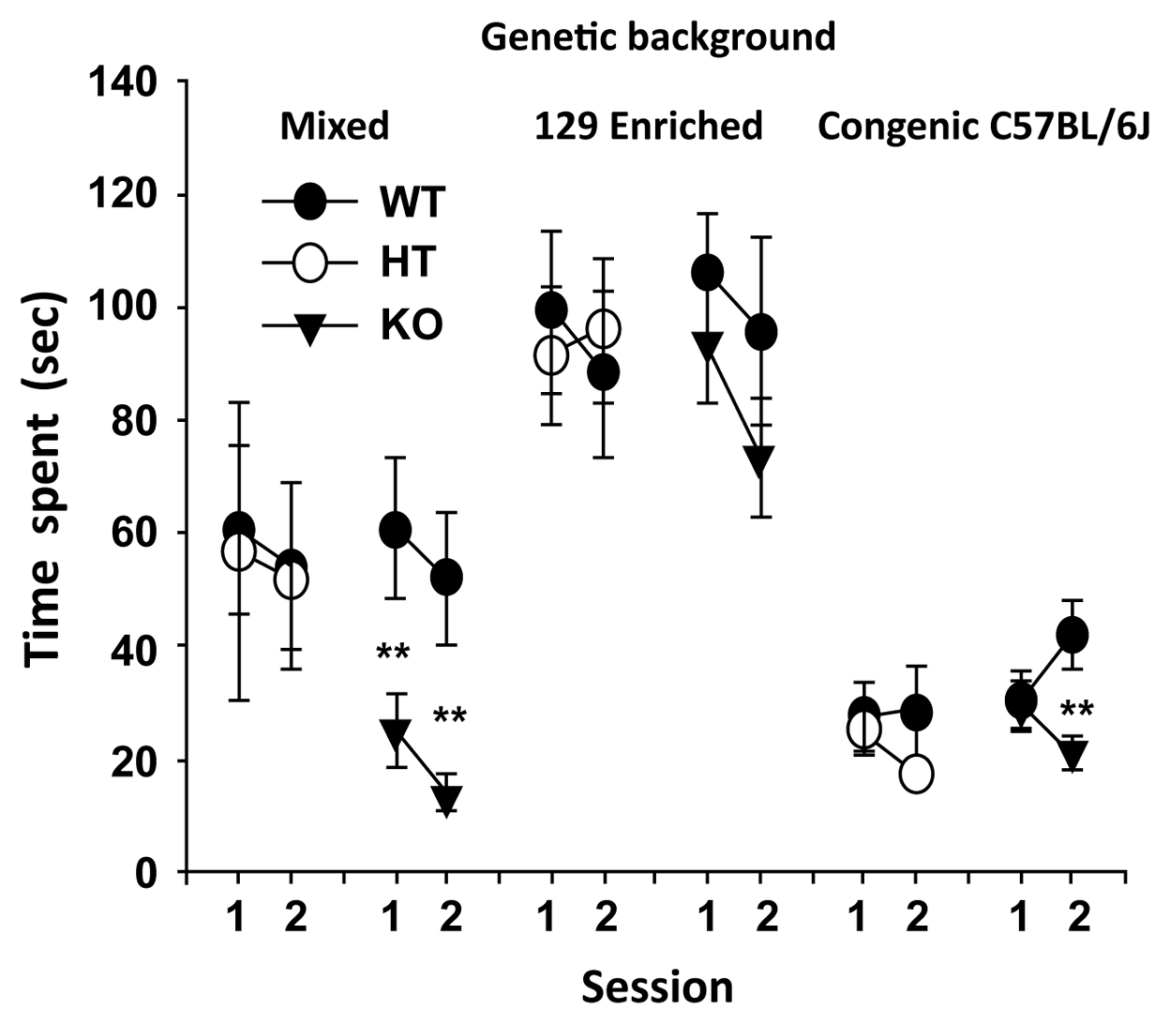
Impact of genetic background on active social interaction in *Sept5* deficient mice. Interaction time (mean ± SEM) spent in active social interaction between mice is shown in two successive 5-min sessions. Asterisks indicate statistically significant differences from WT mice at 1% (**) levels, as determined by Newman-Keuls comparisions. This figure is reproduced from [108,109] with permission of the Oxford Press.

To address the first issue, we tested the impact of *Sept5* deficiency on social interaction on three genetic backgrounds. Active affiliative social interaction was impaired in *Sept5* homozygous (KO) mice with a mixed genetic background of CD1, 129X1/SvJ and 129S1/Sv-p+ Tyr+ Kitl ^SI-J/+^ (Figure 3, Mixed) and with a congenic background with C57BL/6J (Figure 3, Congenic C57BL/6J), but not with a 129S1-enriched genetic background (Figure 3, 129 Enriched) [108]. *Sept5* KO mice were not impaired in other behavioral measures, including working memory and repetitive behavioral trait (spontaneous alternation), PPI, anxiety-related traits, and motor activity [108–110], underscoring a rather selective action of *Sept5* deficiency on symptomatic elements of ASD. Given that *Sept5* deletion is included in 22q11.2 hemizygosity in humans, this gene is likely to contribute to one symptomatic element of ASD. A corollary of this observation is that as long as a gene deficiency causes at least one (not necessarily all) aspect of ASD in a mouse model, a gene should be considered to be a contributory one.

Interestingly, *Sept5* heterozygous mice were not impaired in affiliative social interaction, while 22q11.2 *hemizygosity* is sufficient to induce a high rate of ASD in humans. However, it is not known whether a gene-dose manifests itself similarly in mice and humans. Moreover, *Sept5* heterozygosity is a single gene deficiency, but 22q11.2 hemizygosity carries deficiencies of multiple genes and heterozygosity of other 22q11.2 genes might amplify the impact of *Sept5* heterozygosity in humans.

Our finding offered a plausible explanation for incomplete penetrance, but it did not entirely rule out the possibility that the phenotypic difference between congenic *Sept5* WT and KO mice is caused by allelic heterozygosity instead of –or possibly in addition to—*Sept5* deficiency. A simple estimate based on the number of backcrossings is that our congenic WT and KO mice are homozygous with C57BL/6J alleles at up to 99.8% of loci and the remaining fraction is heterozygous for alleles. However, one often ignored caveat of this estimate is that allelic homozygosity greatly differs at loci linked compared to those not linked to the gene of interest [124]. Even after 10 generations of back-crossing, more alleles from 129 mice are expected to be present at loci near the *Sept5* gene in KO mice than in WT mice. Thus, our observation still does not rule out the possibility that social interaction deficits in congenic KO mice reflect allelic differences rather than *Sept5* deletion. Currently available breeding techniques do not offer a definitive technical option to entirely rule out this possibility [125,126]. There are many mouse models of ASD with non-congenic background. Caution is needed in ascribing a phenotypic difference between mutant and wild type mice to the impact of the mutant gene rather than allelic differences in the genetic background. It would be interesting to observe how the phenotypic expression of other ASD-related genes is modified by genetic background in other mouse models of ASD.

**Figure 4:**
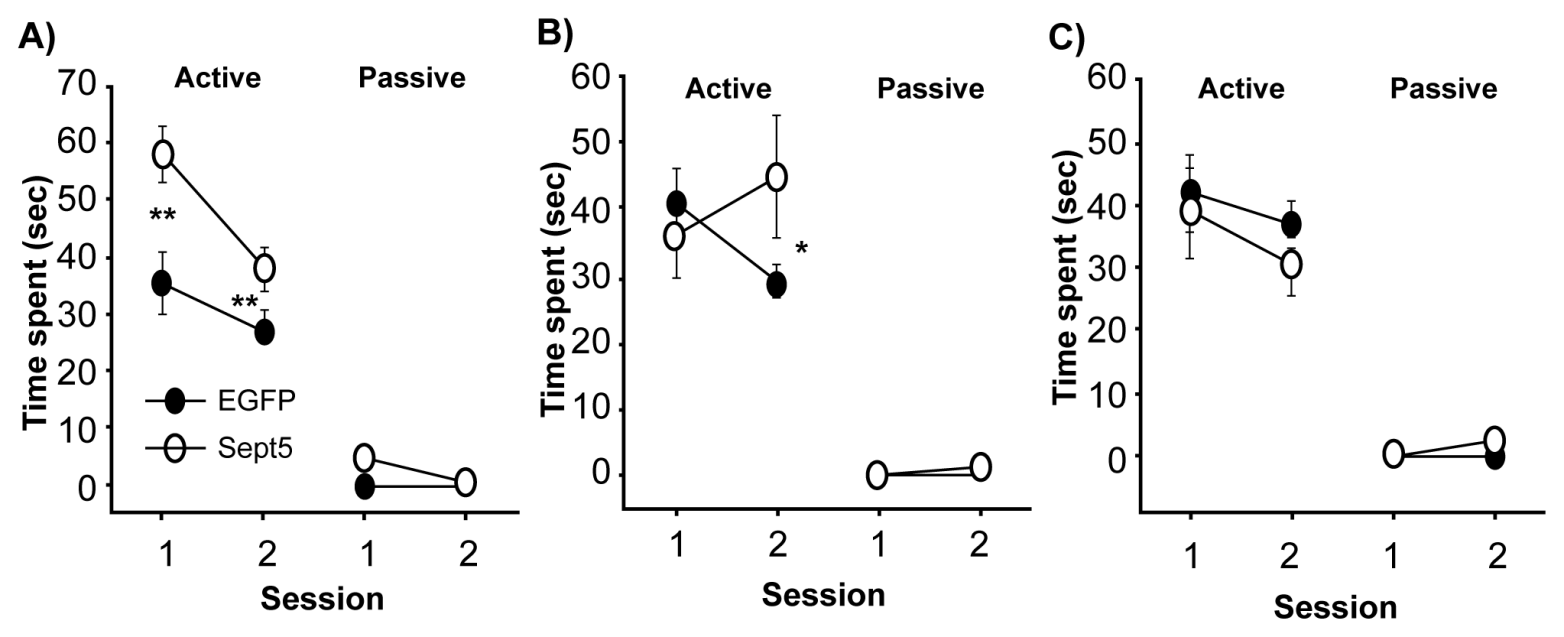
Effects of virally overexpressed Sept5 on active and passive social interaction in the dorsal hippocampus (A), basolateral amygdaloid complex (B), or somatosensory cortex (C). **and*, significant at 1 and 5% level, as determined by Newman-Keuls comparisons. This figure is reproduced from [109] with permission of the Oxford Press.

To address this interpretative caveat and identify brain regions in which *Sept5* levels regulate social interaction, we expressed *Sept5* in selected brain regions at the time of behavioral testing in inbred C57BL/6J mice [109]. We constructed a lentiviral vector carrying *Sept5* and surgically infused it into the brains of C57BL/6J mice, thereby elevating only *Sept5* in distinct brain regions in a congenic genetic background. Compared to control mice that received enhanced green fluorescent protein (EGFP) alone, C57BL/6J mice that received *Sept5-EGFP* over expression in the hippocampus (Figure 4A) and amygdala (Figure 4B) showed increased active, affiliative social interaction. This phenotype was highly selective; *Sept5* overexpression had no effect on reaction to a novel, non-mouse object, olfactory senses, anxiety-related behaviors or motor behavior [109]. Moreover, *Sept5* over-expression in the somatosensory cortex had no effect on social interaction (Figure 4C). Although synaptic alterations in the sensorimotor cortex have been observed in some mutant mouse models of ASD and cortical development has been suggested to be aberrant in ASD, *Sept5* in this cortical region does not seem to have any effect on social interaction.

Our observations indicate that *Sept5* is indeed a determinant of social interaction, but alleles in the genetic background may modulate phenotypic expression of 22q11.2-associated syndromic ASD. Consistent with our mouse phenotype, one child has recently been identified with homozygous deletion of *Sept5* and adjacent *GP1BB*. This child exhibited deficits in motor development, social and emotional function as well as language and speech development [132]. Both parents were heterozygous with no apparent neuropsychiatric phenotypes. Moreover, the impacts of *Sept5* expression in the mouse brain are largely consistent with human studies that underscore the critical roles played by limbic structures in ASD. Structural abnormalities in the hippocampus have been noted in both idiopathic ASD [127,128] and 22q11.2 hemizygous patients [129]. In individuals with 22q11.2 hemizygosity, amygdala activity is anomalous while performing tasks that require social perception [130]. A future challenge is to identify the precise network of structures through which *Sept5* acts as a determinant for social cognition.

One interesting matter is that over-expression of *Sept5* in the hippocampus and constitutive deletion of *Sept5* increases and reduces social interaction, respectively. In an apparent contrast to this observation, clinical observations show that both duplication and hemizygosity induce similar behavioral phenotypes (e.g., social interaction deficits) in humans. However, while over-expression and hemizygosity of Tbx1 have been shown to induce similar cardiovascular phenotypes [131], it is not known if all individual 22q11.2 genes follow the same pattern in behavioral phenotypes. Thus, one possible explanation of our finding is that the dose level of some 22q11.2 genes linearly determines social interaction, but others do so in an inverted U gene-response curve. The phenotypic outcome of 22q11.2 hemizygosity and duplication might reflect the net effect of these additive or opposing phenotypes of multiple genes.

## Conclusion

Systematic searches for 22q11.2 genes that contribute to behavioral phenotypes have identified potential significance of a ~200 kb segment. Data from our genetic mouse models suggest that *Tbx1* and *Sept5* within this ~200 kb region impact multiple or single symptomatic elements of 22q11.2-associated ASD. As Tbx1 is likely to be involved in postnatal neurogenesis [101] and *Sept5* in synaptic contact [133–135] and neurotransmission [121,135,136], these neuronal events should be further explored as potential neuronal substrates for 22q11.2-associated ASD.
